# Supplementary material for: Content-rich biological network constructed by mining PubMed abstracts
Source: BMC Bioinformatics. 2004 Oct 8;5:147. doi: 10.1186/1471-2105-5-147 (PMC528731; doi:10.1186/1471-2105-5-147)
Supplement: Additional File 2 — The original results of the above study (non-essential files are deleted to keep the file size under the limit set by BMC bioinformatics). [file 1471-2105-5-147-S2.bz2 › chilibotAdditionalFile2/dip40/503ID10519421E10670/html/TGFB1_TGFBR3.html]

 


 **TGFB1** and **TGFBR3** 
  
Found 81 abstracts in PubMed, retrieved 40.  
 

 What does Google say? 
 PDF only 
| .edu only 

---

**Interactive relationship** (e.g. stimulation, inhibition, etc)

**Inhibitory relationship**- Betaglycan  [ **TGFBR3** ]  inhibits TGF beta  [ **TGFB1** ]  signaling by preventing type I type II receptor complex formation.  Ref: 11668175 J Biol Chem, 2002
- Abrogation of betaglycan  [ **TGFBR3** ]  attenuates TGF beta  [ **TGFB1** ]  mediated inhibition of embryonic murine lung branching morphogenesis in culture.  Ref: 9739109 Mech Dev, 1998
- We therefore conclude that the abrogation of endogenous betaglycan  [ **TGFBR3** ]  attenuates endogenous autocrine and or paracrine TGF beta  [ **TGFB1** ]  mediated negative regulation of lung organogenesis.  Ref: 9739109 Mech Dev, 1998
- Our data indicate that betaglycan  [ **TGFBR3** ]  can function as a potent inhibitor of TGF beta  [ **TGFB1** ]  signaling by a novel mechanism and provide support for an essential but complex role for proteoglycan co receptors in growth factor signaling.  Ref: 11668175 J Biol Chem, 2002
- We found that recombinant LAP and recombinant soluble betaglycan  [ **TGFBR3** ]  decrease the active form of TGF beta1  [ **TGFB1** ] , measured by ELISA, in a dose dependent manner in a cell free system.  Ref: 12138262 Nephron, 2002
- Betaglycan  [ **TGFBR3** ]  promoter activity was increased by RA and inhibited by the three isoforms of TGF beta  [ **TGFB1** ] .  Ref: 12399463 J Biol Chem, 2003
- We demonstrate that the expression of betaglycan  [ **TGFBR3** ]  in LLC PK1 cells results in inhibition of TGF beta  [ **TGFB1** ]  signaling as measured by reporter gene expression, thymidine incorporation, collagen production, and phosphorylation of the downstream signaling effectors Smad2 and Smad3.  Ref: 11668175 J Biol Chem, 2002
- We demonstrate instead that in LLC PK1 cells, unlike L6 cells, expression of betaglycan  [ **TGFBR3** ]  prevents association between the type I and type II TGF beta  [ **TGFB1** ]  receptors, which is required for signaling.  Ref: 11668175 J Biol Chem, 2002
- Betaglycan  [ **TGFBR3** ]  gene expression decreased in response to serum, but was upregulated by TGF beta1  [ **TGFB1** ]  and unchanged by the other stimulants.  Ref: 11169984 J Cell Physiol, 2001
- The effective dosages of LAP and soluble betaglycan  [ **TGFBR3** ]  for a 50% reduction were approximately 20 and 75 fold of the TGF beta1  [ **TGFB1** ]  concentration, respectively.  Ref: 12138262 Nephron, 2002
- Soluble betaglycan  [ **TGFBR3** ] , prepared as a recombinant protein using the baculoviral expression system, inhibits the actions of TGF beta  [ **TGFB1** ] .  Ref: 11256966 Biochem J, 2001
- Furthermore, abrogation of endogenous betaglycan  [ **TGFBR3** ]  gene expression prevented the characteristic down regulation of cyclin A and surfactant protein C SP C mRNAs by exogenous TGF beta  [ **TGFB1** ]  ligands.  Ref: 9739109 Mech Dev, 1998
- We have demonstrated previously that ectopic expression of a soluble betaglycan  [ **TGFBR3** ] , also known as transforming growth factor TGF beta  [ **TGFB1** ]  type III receptor, can suppress the malignant properties of human carcinoma cells by antagonizing the tumor promoting activity of TGF beta  [ **TGFB1** ]  A.  Ref: 12183427 Cancer Res, 2002
**Stimulatory relationship**- SDS polyacrylamide gel electrophoresis analysis of radiolabeled receptors demonstrated that expression of endoglin or betaglycan  [ **TGFBR3** ]  is associated with an increased TGF beta  [ **TGFB1** ]  binding to the signaling receptor complex.  Ref: 9830054 J Biol Chem, 1998
- In comparison, the expression of betaglycan  [ **TGFBR3** ]  in L6 myoblasts enhances TGF beta  [ **TGFB1** ]  signaling, which is consistent with the published literature.  Ref: 11668175 J Biol Chem, 2002
- A third cell surface receptor, betaglycan  [ **TGFBR3** ] , serves as a co receptor for TGF beta  [ **TGFB1** ]  in some cell types, enhancing TGF beta  [ **TGFB1** ]  mediated signaling.  Ref: 11668175 J Biol Chem, 2002
- Although betaglycan  [ **TGFBR3** ]  TGF beta  [ **TGFB1** ]  type III receptor is known to enhance TGF beta  [ **TGFB1** ]  ligand binding to its type II receptor in murine lung epithelial cell lines, the biological significance of this phenomenon in the process of lung organogenesis is not understood.  Ref: 9739109 Mech Dev, 1998
- ... overexpression of betaglycan  [ **TGFBR3** ]  resulted in an enhanced response to inhibition of cellular proliferation and plasminogen activator inhibitor 1 induced expression in the presence of TGF beta  [ **TGFB1** ] .  Ref: 9830054 J Biol Chem, 1998
**Neutral relationship**- The receptor III or betaglycan  [ **TGFBR3** ]  binds and presents TGF beta1  [ **TGFB1** ]  or beta2 to receptor II.  Ref: 10590020 Pediatr Res, 1999
- Ser544Ala of the murine betaglycan  [ **TGFBR3** ]  cDNA produced a TGF beta  [ **TGFB1** ]  type III receptor devoid of glycosaminoglycan chains.  Ref: 9659379 Biochim Biophys Acta, 1998
- Ligand competition assays indicate that the relative affinities of recombinant soluble betaglycan  [ **TGFBR3** ]  for the TGF beta  [ **TGFB1** ]  isoforms are TGF beta2>TGF beta3>TGF beta1  [ **TGFB1** ] .  Ref: 11256966 Biochem J, 2001
- These results show that betaglycan  [ **TGFBR3** ]  expression is essential for optimal TGF beta  [ **TGFB1** ]  signaling during embryonic lung development.  Ref: 9739109 Mech Dev, 1998
- Our findings suggest that endoglin may modify TGF beta  [ **TGFB1** ]  signalling by interacting with both betaglycan  [ **TGFBR3** ]  and the TGF beta  [ **TGFB1** ]  signalling receptors at physiological receptor concentrations and ratios.  Ref: 10951214 Eur J Biochem, 2000
- The induction of TGF beta  [ **TGFB1** ]  immunoreactivity colocalized with immunoreactive betaglycan  [ **TGFBR3** ]  RIII in activated macrophages, suggesting that LTGF beta activation occurs on the cell surface.  Ref: 9989773 J Cell Physiol, 1999
- In myotubes, up regulated betaglycan  [ **TGFBR3** ]  was also detectable by TGF beta  [ **TGFB1** ]  affinity labeling and immunofluorescence microscopy studies.  Ref: 12399463 J Biol Chem, 2003
- Transfection of this cDNA into COS1 cells resulted in the expression of a membrane proteoglycan that binds TGF beta  [ **TGFB1** ]  and is recognized by antibodies raised against rat betaglycan  [ **TGFBR3** ] .  Ref: 9659379 Biochim Biophys Acta, 1998
- These results suggest that the ALK5 SMAD4 part of the TGFB  [ **TGFB1** ]  signalling pathway is a major target for inactivation in pancreatic carcinomas, that the expression of TGFBR2,  **TGFBR3** , and receptors involved in TGFB  [ **TGFB1** ]  activation are maintained,.  Ref: 11408925 Int J Oncol, 2001
- The anti TGF beta  [ **TGFB1** ]  potency of recombinant soluble betaglycan  [ **TGFBR3** ]  in vitro is 10 fold higher for TGF beta2 than for TGF beta1  [ **TGFB1** ] .  Ref: 11256966 Biochem J, 2001
- TGF beta1  [ **TGFB1** ]  was found in association with latency associated peptide LAP, betaglycan  [ **TGFBR3** ]  and decorin in an isolated dentine matrix preparation.  Ref: 9541223 Eur J Oral Sci, 1998
- The primary structure of murine betaglycan  [ **TGFBR3** ] , also known as transforming growth factor beta TGF beta  [ **TGFB1** ]  type III receptor, was deduced from the nucleotide sequence of a cDNA clone isolated from a heart library.  Ref: 9659379 Biochim Biophys Acta, 1998
- Compared with a commercial pan specific anti TGF beta  [ **TGFB1** ]  neutralizing antibody, recombinant soluble betaglycan  [ **TGFBR3** ]  is more potent against TGF beta2 and similar against TGF beta1  [ **TGFB1** ] .  Ref: 11256966 Biochem J, 2001
- Similarly to the natural soluble betaglycan  [ **TGFBR3** ] , baculoviral soluble betaglycan  [ **TGFBR3** ]  has an equilibrium dissociation constant K d of 3.5 nM for TGF beta1  [ **TGFB1** ] .  Ref: 11256966 Biochem J, 2001
- Betaglycan  [ **TGFBR3** ] , also known as the transforming growth factor beta TGF beta  [ **TGFB1** ]  type III receptor, is a membrane anchored proteoglycan that binds TGF beta  [ **TGFB1** ]  via its core protein.  Ref: 11278442 J Biol Chem, 2001
- Betaglycan  [ **TGFBR3** ]  is an accessory receptor of members of the transforming growth factor beta TGF beta  [ **TGFB1** ]  superfamily, which regulates their actions through ligand dependent interactions with type II receptors.  Ref: 11256966 Biochem J, 2001
- The type III receptor, also known as betaglycan  [ **TGFBR3** ] , binds to the type II receptor and is thought to act solely by presenting the TGF beta  [ **TGFB1** ]  ligand to the type II receptor.  Ref: 11323414 J Biol Chem, 2001
- Betaglycan  [ **TGFBR3** ]  was immunolocalized mainly to the epithelial cells in developing airways, a spatial distribution which overlaps with that of TGF beta  [ **TGFB1** ]  type II receptor.  Ref: 9739109 Mech Dev, 1998
- These results indicate that baculoviral soluble betaglycan  [ **TGFBR3** ]  has the biochemical and functional properties that would make it a suitable agent for the treatment of the diseases in which excess TGF beta  [ **TGFB1** ]  plays a central physiopathological role.  Ref: 11256966 Biochem J, 2001
- Association of TGF beta1  [ **TGFB1** ]  with betaglycan  [ **TGFBR3** ] , decorin and LAP may regulate the availability and biological activity of this growth factor and influence its presentation to the TGF beta  [ **TGFB1** ]  type I and II receptors on odontoblasts.  Ref: 9541223 Eur J Oral Sci, 1998
- The regulation by endoglin of TGF beta  [ **TGFB1** ]  responses seems to reside on the extracellular domain, as evidenced by the functional analysis of two chimeric proteins containing different combinations of endoglin and betaglycan  [ **TGFBR3** ]  domains.  Ref: 9830054 J Biol Chem, 1998
- All together, the present results suggest that betaglycan  [ **TGFBR3** ]  ectodomain is endowed with two bona fide independent ligand binding domains that can perform specialized functions as co receptors of distinct members of the TGF beta  [ **TGFB1** ]  superfamily.  Ref: 11278442 J Biol Chem, 2001

**Non-interactive relationship** (e.g. studied together, co-existance, homology, etc.)

- We compared endoglin distribution with that of TGF beta  [ **TGFB1** ]  receptors type I TbetaR I, type II TbetaR II and betaglycan  [ **TGFBR3** ] .  Ref: 9560476 Cell Tissue Res, 1998
- Endoglin expression on human microvascular endothelial cells association with betaglycan  [ **TGFBR3** ]  and formation of higher order complexes with TGF beta  [ **TGFB1** ]  signalling receptors.  Ref: 10951214 Eur J Biochem, 2000
- These results indicate that recombinant LAP and soluble betaglycan  [ **TGFBR3** ]  suppress the glucose induced overproduction of fibronectin presumably via inhibition of TGF beta1  [ **TGFB1** ]  activity in MCs.  Ref: 12138262 Nephron, 2002
- The major TbetaRIII form, betaglycan  [ **TGFBR3** ] , did not undergo normal posttranslational modification in either of the goblet cell lines and could not bind 125I labeled TGF beta1  [ **TGFB1** ] .  Ref: 9950213 Cell Growth Differ, 1999
- Binding followed by cross linking with 125I TGF beta1  [ **TGFB1** ]  demonstrated that betaglycan  [ **TGFBR3** ]  expressing cells displayed a clear increase about 3.  Ref: 9830054 J Biol Chem, 1998
- These cells can also express TGF beta  [ **TGFB1** ]  type II receptor and betaglycan  [ **TGFBR3** ] .  Ref: 9543637 Curr Eye Res, 1998
- Betaglycan  [ **TGFBR3** ]  in LLC PK1 cells exhibits higher molecular weight glycosaminoglycan GAG chains than in L6 cells, and a GAG betaglycan  [ **TGFBR3** ]  mutant does not inhibit TGF beta  [ **TGFB1** ]  signaling or type I type II receptor association in LLC PK1 cells.  Ref: 11668175 J Biol Chem, 2002
- ... TGF beta  [ **TGFB1** ]  induced inhibition was attenuated to 13 and 26% respectively, in the presence of betaglycan  [ **TGFBR3** ]  antisense oligodeoxynucleotides.  Ref: 9739109 Mech Dev, 1998
- It shares with betaglycan  [ **TGFBR3** ]  a limited amino acid sequence homology and being components of the membrane transforming growth factor beta TGF beta  [ **TGFB1** ]  receptor complex.  Ref: 9830054 J Biol Chem, 1998
- Our data suggested that beta glycan  [ **TGFBR3** ]  TGF beta1  [ **TGFB1** ]  immunosuppressive complexes may contribute to the suppression of accessory cell function in these patients.  Ref: 9543446 AIDS, 1998
- To assess the role of TGF beta1  [ **TGFB1** ]  in the inhibition of accessory cell function in AIDS, antibodies against TGF beta1  [ **TGFB1** ]  or the TGF beta1  [ **TGFB1** ]  type III receptor, beta glycan  [ **TGFBR3** ] , were added to a mixed lymphocyte reaction.  Ref: 9543446 AIDS, 1998
- We therefore studied the expression of UPA, UPAR, IGF2R, ALK5, TGFBR2,  **TGFBR3** , ENG, ALK1,  **TGFB1** , TGFB2, and TGFB3 in a series of 14 pancreatic carcinoma cell lines.  Ref: 11408925 Int J Oncol, 2001
- TGF beta  [ **TGFB1** ]  receptors I, II and III betaglycan  [ **TGFBR3** ]  were present on osteoblasts from both healthy donors and OI patients.  Ref: 11174067 Pathobiology< MedlineTA>Pathobiology, 2001
- Treatment of ovarian cancer HEY cells with 500 nM 5alpha dihydrotestosterone DHT, but not estradiol 17beta or progesterone, for 60 h down regulated the expression of mRNA for TGF beta  [ **TGFB1** ]  receptors I and II TbetaR I and TbetaR II, betaglycan  [ **TGFBR3** ] , and endoglin but had no effect on TGF beta1  [ **TGFB1** ]  mRNA levels.  Ref: 10706107 Cancer Res, 2000
- These findings suggest a role for endoglin in TGF beta  [ **TGFB1** ]  responses distinct from that of betaglycan  [ **TGFBR3** ] .  Ref: 9830054 J Biol Chem, 1998
- Transforming growth factor beta 1  [ **TGFB1** ]  latency associated peptide and soluble betaglycan  [ **TGFBR3** ]  prevent a glucose induced increase in fibronectin production in cultured human mesangial cells.  Ref: 12138262 Nephron, 2002
- Inhibin binding protein InhBP and the transforming growth factor beta TGF beta  [ **TGFB1** ]  type III receptor, beta glycan  [ **TGFBR3** ] , have been identified as putative inhibin coreceptors.  Ref: 11266515 Mol Endocrinol, 2001
- TGF beta  [ **TGFB1** ]  neutralizing antibodies also prevented TGF beta  [ **TGFB1** ]  mediated inhibition of lung branching in culture, supporting the speculation that autocrine paracrine TGF beta  [ **TGFB1** ]  signaling is minimal in the absence of betaglycan  [ **TGFBR3** ] .  Ref: 9739109 Mech Dev, 1998
- 0.01 reduced by the addition of 10 nmol l LAP and 10 nmol l soluble betaglycan  [ **TGFBR3** ]  with no significant change in total active latent TGF beta1  [ **TGFB1** ] .  Ref: 12138262 Nephron, 2002
- All cell populations investigated showed a similar profile of expression of TGF beta  [ **TGFB1** ]  receptors TbetaR I, II and III betaglycan  [ **TGFBR3** ] .  Ref: 10961364 Exp Clin Endocrinol Diabetes, 2000
- Here we show that the type III TGF beta  [ **TGFB1** ]  receptor, betaglycan  [ **TGFBR3** ] , can function as an inhibin co receptor with ActRII.  Ref: 10746731 Nature, 2000
- Endoglin has homology to the type III receptor, betaglycan  [ **TGFBR3** ] , although its exact role in TGF beta  [ **TGFB1** ]  signaling is unclear.  Ref: 10187774 J Biol Chem, 1999
- This study was performed to investigate the effects of transforming growth factor beta1  [ **TGFB1** ]  TGF beta1  [ **TGFB1** ]  latency associated peptide LAP and betaglycan  [ **TGFBR3** ]  on TGF beta1  [ **TGFB1** ]  activity, and on the glucose induced overproduction of fibronectin in cultured human mesangial cells MCs.  Ref: 12138262 Nephron, 2002
- Following abrogation of betaglycan  [ **TGFBR3** ]  expression with antisense oligodeoxynucleotide, embryonic lungs were relatively insensitive to TGF beta  [ **TGFB1** ]  TGF beta2 0.5 ng ml and TGF beta1  [ **TGFB1** ]  20 ng ml, respectively, down regulated lung morphogenesis by 38 and 34% in control cultures.  Ref: 9739109 Mech Dev, 1998
- Here it is shown that on human microvascular endothelial cells, endoglin is co expressed and is associated with betaglycan  [ **TGFBR3** ] , a TGF beta  [ **TGFB1** ]  accessory receptor with which endoglin shares limited amino acid homology.  Ref: 10951214 Eur J Biochem, 2000
- Signal transduction is mediated through the receptor complex, consisting of two active seronine or threonine kinases TGF beta  [ **TGFB1** ]  receptor I and II and the receptor associated proteins betaglycan  [ **TGFBR3** ]  TGF beta  [ **TGFB1** ]  receptor III and endoglin.  Ref: 9808055 Exp Hematol, 1998
- Suppression of protein synthesis reduced TGF beta  [ **TGFB1** ]  binding to types I and II receptors with t1 2 of 2 h and to betaglycan  [ **TGFBR3** ]  with t1 2 of 6 h.  Ref: 8702513 J Biol Chem, 1996
- H50 transcribed the TGF beta  [ **TGFB1** ]  type I and type II receptor R I II, betaglycan  [ **TGFBR3** ] , and endoglin.  Ref: 10555111 AIDS Res Hum Retroviruses, 1999
- Against this background, we studied the cell surface expression of TGF beta  [ **TGFB1** ]  receptors TbetaR I, II and III betaglycan  [ **TGFBR3** ]  on human osteoblast like cells from adult donors, and examined the TbetaR presentation on these cells after a preceding exposure to TGF beta1  [ **TGFB1** ] .  Ref: 10334821 J Endocrinol, 1999
- The most commonly observed receptors are referred to as the type I, type II, and type III betaglycan  [ **TGFBR3** ]  TGF beta  [ **TGFB1** ]  receptors.  Ref: 8702972 J Biol Chem, 1996
- Deletion mutagenesis analysis has revealed two regions of betaglycan  [ **TGFBR3** ]  ectodomain capable of binding TGF beta  [ **TGFB1** ]  one at the amino terminal half, the endoglin related region López Casillas, F.  Ref: 11278442 J Biol Chem, 2001
- We found 11 single nucleotide polymorphisms and one triple nucleotide insertion in the cDNA of the human transforming growth factor beta TGF beta  [ **TGFB1** ]  III receptor gene  **TGFBR3**  located on 1p33 p32, encoding beta glycan  [ **TGFBR3** ] , a component of the TGF beta  [ **TGFB1** ]  receptor system.  Ref: 10944857 J Hum Genet, 2000
- The addition of blocking antibodies against either TGF beta1  [ **TGFB1** ]  or beta glycan  [ **TGFBR3** ]  restored the T cell stimulatory function to accessory cells from these patients.  Ref: 9543446 AIDS, 1998
- Leukemic BCPs were positive for gene transcription of TGF beta  [ **TGFB1** ]  9 9, the TGF beta  [ **TGFB1** ]  receptor I 9 9, the TGF beta  [ **TGFB1** ]  receptor II 6 6, betaglycan  [ **TGFBR3** ]  5 6, and endoglin 6 6.  Ref: 9808055 Exp Hematol, 1998
- For this purpose we chose the TGF beta  [ **TGFB1** ]  accessory receptor, betaglycan  [ **TGFBR3** ] , since genetic and biochemical evidence showed that betaglycan  [ **TGFBR3** ]  is not a substrate of the shedding system.  Ref: 9202036 J Biol Chem, 1997
- Recombinant LAP and soluble betaglycan  [ **TGFBR3** ]  also inhibited a recombinant TGF beta1  [ **TGFB1** ]  stimulated increase in fibronectin production in MCs.  Ref: 12138262 Nephron, 2002
- Because of its potential use as an anti TGF beta  [ **TGFB1** ]  therapeutic agent, we have purified and characterized baculoviral recombinant soluble betaglycan  [ **TGFBR3** ] .  Ref: 11256966 Biochem J, 2001
- Forced expression of betaglycan  [ **TGFBR3** ]  in CC myoblasts increases their responsiveness to TGF beta2, suggesting that it performs a TGF beta  [ **TGFB1** ]  presentation function in this cell lineage.  Ref: 12399463 J Biol Chem, 2003
- Confocal microscopy of metabolically active macrophages incubated with antibodies to TGF beta  [ **TGFB1** ]  and betaglycan  [ **TGFBR3** ]  RIII prior to fixation supported the localization of activation to the cell surface.  Ref: 9989773 J Cell Physiol, 1999
